# Paxlovid-Induced Tacrolimus Toxicity in the Treatment of COVID-19: A Case Report

**DOI:** 10.7759/cureus.35489

**Published:** 2023-02-26

**Authors:** Stephanie Michael, Rachel Heilbronner, Christopher M Lloyd, Howard W Levitin

**Affiliations:** 1 Emergency Medicine, OhioHealth Doctors Hospital, Columbus, USA; 2 Pharmacology and Therapeutics, OhioHealth Doctors Hospital, Columbus, USA

**Keywords:** pandemic emergency medicine, geriatrics and internal medicine, drug-drug interactions, paxlovid, covid-19

## Abstract

Paxlovid^TM^ (nirmaltrelvir/ritonavir) received emergency use authorization from the Food and Drug Administration (FDA) in December 2021 to treat coronavirus disease 2019 (COVID-19). Given the actions of Paxlovid on cytochrome P450-3A4 (CYP3A4) enzymes, it is imperative to check for potential drug-drug interactions before prescribing. We describe a case in which the common emergency department presentation of generalized weakness was found to be caused by interactions between Paxlovid and a patient’s home medications resulting in tacrolimus toxicity.

## Introduction

Emergency physicians care for patients with vague and non-specific complaints on a daily basis [1]. A concern for generalized weakness in an elderly patient prompts consideration of a wide variety of etiologies including but not limited to infection, electrolyte abnormalities, dehydration, cardiovascular disease, ischemia, endocrine dysfunction, and anemia. Often overlooked is the possibility of medication side effects, drug-drug interactions, and polypharmacy in this high-risk population. Between December 23, 2021, and May 21, 2022, over 800,000 Paxlovid prescriptions were dispensed based on data from the United States Health and Human Services and it continues to be utilized for high-risk individuals testing positive for COVID-19 [2]. Our case emphasizes the importance of reviewing a patient’s medication list (prescribed, over-the-counter medicines, herbs and vitamins), specifically as it relates to a new therapeutic such as Paxlovid, and any new medications that have been prescribed when evaluating a patient with generalized weakness. 

## Case presentation

A 79-year-old male with a known history of essential hypertension and a liver transplant 10 years previously presented to the emergency department (ED) with a chief complaint of generalized weakness. The patient reported nine days of symptoms, including mild diarrhea, nausea with retching, and dry cough. He tested positive for COVID-19 shortly after the start of this recent illness. He was concerned that he was not improving as expected and suspected dehydration played a role in his delayed recovery. The patient reported a diminished appetite, could only tolerate minimal fluids and felt generally weak with reduced urine output over the last few days. His ongoing diarrhea, consisting of loose brown stools, was most distressing for the patient. He noted his dry cough had not worsened and only occasionally included the production of clear sputum. The patient denied abdominal or chest pain, shortness of breath, headache or vision changes, focal weakness or paresthesia, urinary frequency or urgency, or bloody stools. No recent travel or antibiotic use was reported.

The physical exam revealed a fatigued-looking older man who was awake, alert, and oriented (x3), nontoxic in appearance, and without acute distress. The gentleman's physical exam that included the head, eyes, ears, nose and throat only demonstrated dry oral mucous membranes, while his cardiac evaluation revealed a regular rate and rhythm without a murmur. His pulmonary examination was unremarkable, though mildly tachypneic. The abdominal exam revealed prior surgical scars, a lack of tenderness with palpation, and no mass or hernia was appreciated. No jaundice, rash, pallor, or evidence of recent trauma was noted on his skin. Neurologically he was without focal deficits, and his gross motor function was intact. 

Diagnostics section

Basic laboratory testing was obtained to help differentiate the etiology of his general sense of weakness (Table 1). A complete blood count was notable for slight anemia with a hemoglobin of 12.7 g/dl, down from his baseline of 14.5 g/dl. His basic metabolic profile revealed hyponatremia with a sodium of 129 mmol/L, mild hyperkalemia with a potassium of 6.0 mmol/L, bicarbonate of 17 mmol/L, glucose of 114 mg/dl, blood urea nitrogen (BUN) of 82 mg/dl, creatinine of 2.59 mg/dl from a baseline of 1.19 mg/dl with a reduced glomerular filtration rate (GFR) of 24 ml/min. His elevation in creatinine and BUN was concerning for acute kidney injury (AKI) with a BUN to creatinine ratio of 31, insinuating prerenal azotemia.

**Table 1:** Laboratory Workup H: high; L: low; WBC: white blood cells; RBC: red blood cells

Lab	Value	Reference Range
WBC Count	5.54	4.5- 11.00 K/mcL
Absolute Neutrophils	3.14	1.7-7.00 K/mcL
RBC Count	4.23	4.5-5.9 M/mcL
Hemoglobin	12.7	13.5-17.5 g/dl
Hematocrit	38.5	41-53%
Mean Corpuscular Volume	91	80-100 fL
Platelet Count	130 L	150-400 K/mcL
Sodium	129 L	135-145 mmol//L
Potassium	6.0 H	3.5-5.1 mmol/L
Chloride	101	98-108 mmol/L
Bicarbonate	17	21-32 mmol/L
Anion Gap	17	10-20 mmol/L
Glucose	114 H	65-99 mg/dL
Blood Urea Nitrogen (BUN)	82 H	8-25 mg/dL
Creatinine	2.59 H	0.8-1.30 mg/dL
Estimated Glomerular Filtrate Rate (eGFR)	24 L	>60 ml/min/1.73m^2
BUN/Creatinine Ratio	31.7 H	10.0-20.0
Calcium	8.5	8.4-10.2 mg/dL
Tacrolimus Level	26.6 H	5-15 ng/ml

A chest X-ray was obtained to rule out pneumonia in the setting of his recent COVID-19 infection which was negative for focal consolidation, pleural effusion, or other acute intrathoracic abnormality. 

Since the degree of AKI was not consistent with the anticipated fluid loss from one to two loose stools per day, additional medical history was sought. The follow-up interview revealed that the patient recently completed a five-day course of Paxlovid for his COVID-19 infection. Additionally, he has taken two immunosuppressant medications daily, tacrolimus and mycophenolate, to prevent organ transplant rejection. This detail was either unknown or underappreciated by the treating physician who prescribed Paxlovid during his previous ED visit nine days prior. With this new information, other causes of AKI were considered, including intrinsic renal dysfunction from Paxlovid-induced tacrolimus toxicity and other post-renal causes. Bedside renal ultrasound did not reveal evidence of hydronephrosis and the ultrasound of a non-post void bladder demonstrated only 356 ml of urine. Based on these ultrasound findings, a post-renal cause for his AKI seemed less likely, thereby elevating the concern for an intrinsic etiology for his renal dysfunction.

Drug-drug interaction research in addition to consultation with the ED pharmacist revealed that the combination of Paxlovid and tacrolimus results in supratherapeutic levels of the latter. This can lead to tacrolimus toxicity which most commonly presents as acute renal dysfunction. This problem was likely compounded by the patient's recent gastrointestinal fluid losses and poor oral intake. The patient required admission to the hospital for management of his AKI while awaiting a tacrolimus blood level, ultimately resulting at 26.6 ng/mL (normal range 5 - 15 ng/mL). Chart review revealed this patient’s tacrolimus levels within the therapeutic range at 5.8 - 6.7 ng/mL in the preceding three months, demonstrating the novelty in his supratherapeutic level on this encounter.

## Discussion

Paxlovid is an oral combination antiviral medication recently approved by the FDA for the treatment of high-risk individuals who recently tested positive for COVID-19. Treatment of unvaccinated, symptomatic, high-risk COVID-19 patients with Paxlovid resulted in an 89% lower risk of disease progression to severe COVID-19 compared to placebo without significant safety concerns [2]. Paxlovid includes two antiviral drugs, nirmatrelvir and ritonavir, both protease inhibitors originally developed for other clinical applications including severe acute respiratory syndrome (SARS) in 2002 (nirmatrelvir) and human immunodeficiency virus (HIV-1) (ritonavir). Both are also potent inhibitors of the cytochrome P450-3A4 (CYP3A4) enzyme responsible for metabolizing multiple classes of medications. Additionally, ritonavir reduces the breakdown of nirmatrelvir, thereby increasing its plasma concentration for efficacy, while also delaying its clearance [3,4].

When the severe acute respiratory syndrome coronavirus 2 (SARS-CoV-2) virus invades an infected cell, it uses the host’s translational apparatus to create the proteins needed to survive and replicate. That biochemical process translates the viral genome into two large polyproteins that are then cleaved into smaller functional units by two protease enzymes. By blocking the SARS-CoV-2 virus’s main polyprotein protease enzyme, nirmatrelvir inhibits the virus’s ability to produce any functional proteins and therefore becomes unable to replicate [5].

By delaying nirmatrelvir’s breakdown in the liver, ritonavir allows for higher drug concentrations and delayed elimination. While this could be favorable for therapeutic use in the management of viral infections such as COVID-19, the effect on P450 is potentially problematic in patients who are also prescribed a broad range of medications that utilize this enzymatic process. This wide variety of medications includes some anticoagulants, anticonvulsants, corticosteroids, pethidine, amiodarone, flecinide, colchicine, clozapine, lovastatin, simvastatin, sildenafil, midazolam and certain immunosuppressants [6].

In this case, the patient was 10 years status-post liver transplant on two immunosuppressant medications common in solid organ transplant recipients, tacrolimus and mycophenolate. Irreversible inhibition of the P450 system by ritonavir resulted in suppressed metabolism and supratherapeutic levels of tacrolimus. Coadministration of these two drugs increases the risk of tacrolimus toxicity 57-fold [7]. Such toxicity may result in nephrotoxicity, the development of hemolytic-uremic syndrome, and neurotoxicity [8,9]. In addition, patients may experience increased risk of infection, burning or tingling of the mouth, hands or feet, insomnia, headaches, hyperglycemia, hypertension, nausea and vomiting, hyperkalemia, decreased magnesium, tremors, diarrhea, loss of appetite, and hair loss [10]. 

The University of Liverpool COVID-19 Drug Interactions database (covid19-druginteractions.org) strongly recommends against using Paxlovid in those individuals on tacrolimus due to this interaction and subsequent risks. The National Institutes of Health recommends contacting the patient’s transplant specialist prior to prescribing Paxlovid to facilitate appropriate monitoring of tacrolimus to reduce toxicities [11]. In patients who are deemed appropriate for Paxlovid therapy following informed consent and transplant team buy-in, tacrolimus may be held, adjusted, or closely monitored for weeks prior to resuming pre-Paxlovid dosing [12].

## Conclusions

Paxlovid (nirmatrelvir and ritonavir) is approved for the outpatient treatment of mild-to-moderate COVID-19 in patients 12 years of age and older who are at high risk for disease progression, including hospitalization and death. High-risk individuals are defined as those with a history of cancer, chronic disease, diabetes, immunodeficiencies, obesity, mental health disorders, pregnancy, or organ transplant. These patients are typically on various medications that predispose them to drug-drug interactions when started on Paxlovid. In this case, initiation of Paxolvid in a patient on lifelong tacrolimus resulted in the patient developing tacrolimus toxicity and acute renal injury. This could have been prevented with a more in-depth review of this patient’s medication history prior to prescribing Paxlovid. Clinicians must always review a patient’s current medication list (prescribed and over-the-counter) and history of recent illness before attributing non-specific complaints to a self-limited viral infection or dehydration. 
